# Editorial: Single-cell analysis on the pathophysiology of autoimmune diseases

**DOI:** 10.3389/fimmu.2024.1451354

**Published:** 2024-07-02

**Authors:** Shiang-Jong Tzeng, InKyeom Kim, Kuang-Hui Sun

**Affiliations:** ^1^ Graduate Institute of Pharmacology, College of Medicine, National Taiwan University, Taipei, Taiwan; ^2^ Department of Pharmacology, School of Medicine, Kyungpook National University, Daegu, Republic of Korea; ^3^ BK21 Plus Kyungpook National University (KNU) Biomedical Convergence Program, School of Medicine, Kyungpook National University, Daegu, Republic of Korea; ^4^ Cardiovascular Research Institute, School of Medicine, Kyungpook National University, Daegu, Republic of Korea; ^5^ Department of Biotechnology and Laboratory Science in Medicine, National Yang Ming Chiao Tung University, Taipei, Taiwan

**Keywords:** autoimmune disease, single-cell RNA sequencing (scRNA-seq), transcriptomics, epigenomics, mass cytometry, CITE-seq, pathophysiology, therapy

Autoimmune diseases are characterized by a complex interplay of diverse immune cell types, each fulfilling unique roles and functions. Traditional bulk analysis methods tend to average signals across mixed cell populations, thereby obscuring the contributions of individual cell types. In contrast, single-cell analysis enables the precise identification of specific cell subsets that play pivotal roles in driving disease pathogenesis and progression, thus providing novel insights into autoimmune disorders. Moreover, this approach facilitates the detection and characterization of rare cells with significant pathogenic potential, enhancing our understanding of their functional roles and contributions to disease mechanisms. By scrutinizing the transcriptome, proteome, or epigenome of individual cells, researchers can uncover distinct gene expression patterns, protein profiles, and regulatory mechanisms specific to particular cell types or disease states. This comprehensive molecular profiling not only aids in identifying biomarkers crucial for diagnosis, prognosis, and the development of therapeutic targets, but also reveals intricate details that might be overlooked by bulk analysis methods.

In this Research Topic on single-cell analysis, we present a collection of 15 papers exploring various aspects of autoimmune diseases, including sequencing methods, genetic and epigenetic contributions, molecular mechanisms and pathogenesis, biomarkers, and therapeutic implications. By examining these factors at the single-cell level, we aim to enhance our understanding of autoimmune diseases and highlight new potential diagnostics and therapeutics.

Three studies are related to single-cell methods. The cellular indexing of transcriptomes and epitopes by sequencing (CITE-seq) enables the identification cell types through the expression of surface markers not captured by single-cell RNA sequencing (scRNA-seq), while simultaneously quantifying gene and protein surface expressions (1). Colpitts et al. used paired CITE-seq and flow cytometry to characterize resident immune cells in human islets that would have been difficult to detect based on mRNA expression alone. Their analysis revealed valuable insights into the cellular dynamics within human islets. In analytic methods, Fritz et al. reviewed advancements in single-cell computational machine learning to characterize the heterogeneity of fibroblasts and uncover novel fibroblast-macrophage interactions driving immune-mediated inflammatory diseases such as rheumatoid arthritis (RA) and psoriasis. Their review unraveled promising therapeutic targets, including CSF1R, PDGFR, and EGFR, which could lead to new treatment strategies for these diseases. Balog et al. employed single-cell mass cytometry (or CyTOF, cytometry by time-of-flight) (2) to comprehensively characterize and compare 17 immune cell populations in peripheral blood samples from healthy controls, treatment-naive patients with RA, systemic sclerosis, and systemic lupus erythematosus (SLE). They utilized 34 markers and analyzed 59 scatter plots to elucidate disease-specific population frequencies and expression patterns of immune cells. Their study represents a valuable single-cell data resource that enhances our understanding of the immune cell landscape in these autoimmune diseases.

Four studies have employed single-cell analysis to investigate SLE. Patients with lupus nephritis (LN) are susceptible to irreversible kidney damage or failure. Daamen et al. conducted scRNA-seq on the kidneys of female lupus-prone NZM2328 mice at acute, transitional, and chronic stages of the disease. They compared gene expression profiles between these mice and human LN patients to elucidate molecular mechanisms driving LN progression. Utilizing unsupervised gene co-expression network analysis, such as MEGENA (3), they characterized molecular profiles correlating with disease severity. Their gene expression analysis offers a method to stage LN in lupus-prone mice and translate these findings to human LN patients. Additionally, Daamen et al. explored the heterogeneity of splenic IL-10 producing regulatory B cells (Breg) across disease stages in lupus-prone mice, revealing several insights from scRNA-seq analysis: active disease is marked by a loss of marginal zone-lineage Bregs, an increase in plasmablast/plasma cell-lineage Bregs, and overall elevation in inflammatory gene signatures. These findings underscore the dynamic changes in Breg populations and their roles in lupus pathogenesis. Filia et al. performed a comprehensive analysis of hematopoietic stem and progenitor cells (HSPCs) in SLE patients. Their study revealed a decrease in non-proliferating early progenitors with an interferon (IFN) signature, implicated in the functional loss and depletion of HSPCs. The data suggest that HSPCs act as sensors of IFN-related inflammatory signals, initiating the inflammatory processes characteristic of SLE. Of interest, Cui et al. explored the shared mechanisms between SLE and primary Sjögren’s syndrome (pSS) by analyzing shared hub genes, related pathways, and transcription factors (TFs) in scRNA-seq datasets from the peripheral blood of patients with SLE and pSS. They identified IFI44L, ISG15 and ITGB2 as shared hub genes involved in the IFN response and ITGB2 signaling pathways. Additionally, they found that STAT1 and IRF7 are common TFs associated with monocytes and dendritic cells (DCs) in both SLE and pSS patients.

Three single-cell studies have investigated autoimmune disorders affecting the skin. Kim et al. analyzed human psoriasis lesions before and after 12 weeks of systemic IL-17A blockade using a multi-omics approach that integrated immune cell-enriched scRNA-seq, microarray, and immunohistochemistry data. They discovered that systemic IL-17A inhibition not only blocked the entire IL-23/T17 cell axis in T cells, DCs, and keratinocytes, but also promoted regulatory gene expression in regulatory DCs present in human psoriasis skin, such as BDCA-3 (THBD) and DCIR (CLEC4A). Gao et al. explored the epigenetic pathogenesis of psoriasis by analyzing the involvement of long non-coding RNAs (lncRNAs) known to participate in immune regulation. They conducted an analysis for differentially expressed lncRNAs, co-regulated gene patterns, and GO-bioprocess enrichment to identify lncRNAs that modulate cellular inflammation in psoriasis at the single-cell level. Their study highlights the therapeutic potential of lncRNAs in managing this disease. Epidermolysis bullosa acquisita (EBA) is a chronic autoimmune disorder characterized by subepidermal blistering of the skin and mucous membranes. The condition arises from antibodies (Abs) targeting type VII collagen, the primary constituent of anchoring fibrils responsible for connecting the basement membrane to dermal structures (4). Guerrero-Juarez et al. performed scRNA-seq of whole blood and skin dissociates to characterize the transcriptome of perturbed neutrophils in patients with EBA. Their findings revealed that the upregulation of C-type lectin receptors (Clec4n, Clec4d, and Clec4e) is a hallmark of activated dermal neutrophil populations. However, despite this upregulation, the individual contribution of these genes to the pathogenesis of EBA was found to be dispensable. This highlights the complexity of EBA pathogenesis and underscores the need for further research to uncover critical molecular targets for therapy.

Two studies are related to RA. Methylation of adenosine at N6 position (m6A) is mediated by distinct enzymes and occurs on both coding and non-coding RNAs (5). Geng et al. investigated the m6A methylation regulators in RA. They identified two m6A methylation regulators, IGF2BP3 and YTHDC2, as significant biomarkers for RA. Using consensus feature selection from four methods, these biomarkers were found to predict RA diagnosis with high accuracy. Pathway and network analysis revealed a novel role for IGF2BP3 in M1 macrophage polarization during the progression of RA, offering new strategies for early diagnosis and targeted therapy. In a brief report on a case of COVID-19 vaccination, Ishikawa et al.’s scRNA-seq analysis identified a distinct monocyte population with RA signatures, such as cathepsin L (CTSL) and CXCL8, in peripheral blood during the acute phase of encephalitis (day 3). It remains unclear whether this specific classical monocyte population is commonly observed in COVID-19 vaccination-related CNS diseases or if it reflects the enhanced dysregulated immunity unique to each specific disease. Further studies are needed to determine the generalizability of these findings across different cases of vaccination-related CNS diseases.

Anti-N-methyl-D-aspartate receptor encephalitis (anti-NMDARE) is a rare autoimmune disease characterized by Abs against the glutamate receptor N1 (GluN1) subunit of NMDAR (6). Using scRNA-seq, Jiang et al. observed that patients in the acute phase expressed high levels of DC_CCR7 in peripheral myeloid cells. DC_CCR7 is known to play crucial roles in T-cell activation, differentiation, and the expansion of IgG-producing B cells. This finding implies that DC_CCR7 may contribute to lymphocyte activation during the acute stage of anti-NMDARE, highlighting its potential significance in the disease’s pathogenesis.

Lastly, the topic editor Kim et al.’s group addressed the critical role of immune cells in the development of hypertension (7). They performed scRNA-seq on peripheral blood and lamina propria cells from salt-sensitive male rats receiving a high-fructose solution. Their study pointed out a pivotal role for the upregulation of IFN pathway in B cells in the development of hypertension. This suggests a potential autoimmune factor contributing to the pathogenesis of fructose-induced hypertension in the intestine. Furthermore, their findings indicate that targeting B cells could be a potential intervention strategy to reduce blood pressure in individuals with fructose-induced hypertension, highlighting a novel therapeutic approach in managing this condition.

In conclusion, the findings presented here contribute significantly to identifying pathogenic cell populations, elucidating their interactions, and deciphering the regulatory mechanisms at play. The precision achieved in understanding disease mechanisms holds promise for designing more effective treatments tailored to meet the individual needs of patients. This marks a significant advancement towards personalized medicine in the field of autoimmune disease research, paving the way for innovative therapeutic strategies that could greatly improve patient outcomes.

## Author contributions

S-JT: Writing – original draft, Writing – review & editing. IK: Writing – review & editing. K-HS: Writing – review & editing.

## References

[1] Stoeckius M Hafemeister C Stephenson W Houck-Loomis B Chattopadhyay PK Swerdlow H. Simultaneous epitope and transcriptome measurement in single cells. Nat Methods. (2017) 14:865–8. doi: 10.1038/nmeth.4380 PMC5669064 28759029

[2] Spitzer MH Nolan GP. Mass cytometry: single Cells, many Features. Cell. (2016) 165:780–91. doi: 10.1016/j.cell.2016.04.019 PMC4860251 27153492

[3] Song WM Zhang B. Multiscale embedded gene co-expression network analysis. PloS Comput Biol. (2015) 11:e1004574. doi: 10.1371/journal.pcbi.1004574 26618778 PMC4664553

[4] Kridin K Kneiber D Kowalski EH Valdebran M Amber KT. Epidermolysis bullosa acquisita: A comprehensive review. Autoimmun Rev. (2019) 18:786–95. doi: 10.1016/j.autrev.2019.06.007 31181325

[5] Sommer S Lavi U Darnell JE Jr. The absolute frequency of labeled N-6-methyladenosine in HeLa cell messenger RNA decreases with label time. J Mol Biol. (1978) 124:487–99. doi: 10.1016/0022-2836(78)90183-3 712844

[6] Zhao X Teng Y Ni J Li T Shi J Wei M. Systematic review: clinical characteristics of anti-N-methyl-D-aspartate receptor encephalitis. Front Hum Neurosci. (2023) 17:1261638. doi: 10.3389/fnhum.2023.1261638 38053649 PMC10694196

[7] Drummond GR Vinh A Guzik TJ Sobey CG. Immune mechanisms of hypertension. Nat Rev Immunol. (2019) 19:517–32. doi: 10.1038/s41577-019-0160-5 30992524

